# Research Progress on Flavor Differences and the Formation Mechanism of Traditional Chinese Cereal Vinegar

**DOI:** 10.3390/foods14132263

**Published:** 2025-06-26

**Authors:** Jiayan Zhang, Xuefen Bian, Yansheng Zhao, Ying Zhu, Juan Bai, Songtao Fan, Xiang Xiao

**Affiliations:** School of Food and Biological Engineering, Jiangsu University, Zhenjiang 212013, China; jiayanzhang1988@163.com (J.Z.); bxf20010113@163.com (X.B.); zhaoys@ujs.edu.cn (Y.Z.); ying307@126.com (Y.Z.); 1000005134@ujs.edu.cn (J.B.); fansongtao@ujs.edu.cn (S.F.)

**Keywords:** cereal vinegar, flavor differences, influencing factors, formation mechanism

## Abstract

Cereal vinegar represents a significant traditional vinegar in China. This paper conducts an in-depth exploration, drawing on literature and research, into the raw materials, brewing processes, and flavor profiles of cereal vinegars, including wheat vinegar, sorghum vinegar, and rice vinegar. The research on key flavor compounds focused on organic acids, amino acids, and volatile flavor compounds. This paper revealed their types, variations in content, and specific contributions to the flavor profiles. Different types of vinegar exhibit characteristic volatile flavor compounds. The effects of key factors, including raw materials, fermentation processes, environments, and starters, on cereal vinegar flavor were further examined. The key mechanisms underlying flavor formation were investigated using multi-omics technology. Current research on the flavor formation mechanisms of cereal vinegar remains inadequate, and product diversity lags behind fruit vinegar categories. Given rising consumer emphasis on food health, significant opportunity exists to explore cereal vinegar’s health properties and develop novel functional varieties. This study aims to provide a theoretical foundation for enhancing cereal vinegar quality, fostering product innovation, exploring its flavor and health value, and advancing the preservation and innovation of traditional Chinese cereal vinegar.

## 1. Introduction

By utilizing the fermentation environment (temperature, humidity, water content, oxygen levels, etc.) and employing traditional fermentation techniques, people enable different raw materials (such as vegetables and fruits) to be transformed by microorganisms into fermented foods with distinctive flavors [1,2,3,4]. Cereal vinegar is an acidic condiment produced through microbial fermentation, primarily involving acetic acid bacteria and lactic acid bacteria. etc. It exhibits distinctive sensory properties and nutritional benefits. With a history spanning thousands of years in China, it plays a significant role in the daily diet [5,6]. Traditional Chinese cereal vinegar is produced from sorghum, rice, glutinous rice and other grains. The process involves yeast fermentation to produce ethanol and carbon dioxide, followed by acetic acid fermentation primarily driven by acetic acid bacteria. This forms the unique flavor of cereal vinegar. Additionally, cereal vinegar offers various nutritional benefits, including demonstrated antibacterial properties, as well as potential roles in lipid regulation and weight management. These attributes contribute to its high consumer appeal [7].

In recent years, with the continuous advancement of food analysis technology, the research on the differences of flavor compounds and their formation mechanisms in traditional Chinese cereal vinegar has been gradually deepened. The flavor of cereal vinegar comprises diverse components, including organic acids, amino acids, volatile aroma components, and a variety of functional substances [8]. Studies have revealed distinct variations in both the composition and content of flavor compounds among different types of traditional Chinese cereal vinegars, exemplified by Zhenjiang aromatic vinegar (ZAV), Shanxi aged vinegar (SAV), and Sichuan Baoning bran vinegar (SBV) [9]. These differences are primarily manifested in the types and proportions of organic acids, amino acids, sugars, polyphenols, flavonoids, alcohols, esters, aldehydes, ketones, and other compounds. This further gives each cereal vinegar its unique flavor characteristics. Regarding the formation mechanisms, research indicates that the composition of raw materials, regional environment, and specific fermentation techniques collectively shape the flavor profile of cereal vinegar [10]. Microbial community metabolism plays a key role in the brewing process of cereal vinegar. The macromolecular substances, such as carbohydrates and proteins in raw materials, are decomposed and biotransformed into diverse flavor compounds through intricate metabolic pathways. In addition, environmental factors such as temperature, humidity, oxygen supply during fermentation, and aging time also exert a significant influence on the generation and accumulation of flavor compounds [11]. However, there is still a deficiency in the comprehensive understanding of the flavor distinctions and formation mechanisms of traditional Chinese cereal vinegar. Compared with fruit vinegar, the variety of traditional Chinese cereal vinegar is not rich enough [12,13,14].

This review summarizes the existing information on Chinese cereal vinegar and highlights the main challenges. These includes the insufficient depth of research on the mechanism of flavor formation, as well as the limited diversity of cereal vinegar products compared to fruit vinegar. Based on this review, future research can further reveal the metabolic pathways that control the synthesis of flavor compounds. By combining the microorganisms, genes, enzymes, etc. in the formation pathways with key influencing factors, the key flavor substances can be targetedly regulated to develop innovative cereal vinegar varieties. This strategy is expected to improve product quality, meet the changing consumer demands, promote industry development, and expand the application scope of various grains.

## 2. Types of Traditional Chinese Cereal Vinegar

**Figure 1:**
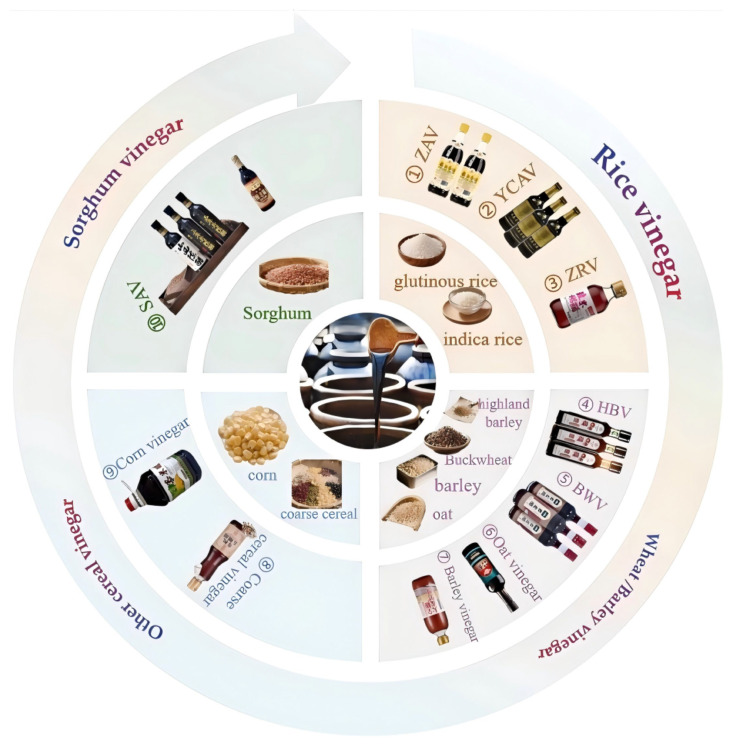
A summary chart of Chinese cereal vinegar varieties and Raw materials. ①: ZAV: Zhenjiang aromatic vinegar; ②: YCAV: Yongchun aged vinegar; ③: ZRV: Zhejiang Rosy vinegar; ④: HBV: Highland barley vinegar; ⑤: BWV: Buckwheat vinegar; ⑥: Oat vinegar; ⑦: barley vinegar; ⑧: Coarse cereal vinegar; ⑨: Corn vinegar; ⑩: SAV: Shanxi aged vinegar.

Traditional Chinese cereal vinegar has a history of thousands of years and is an important part of China’s cultural heritage [15]. According to the different fermentation substrates, it can be divided into cereal vinegar, fruit vinegar [16]. Traditional Chinese cereal vinegar production utilizes grains as the primary raw material. It employs distinctive open fermentation process, involving a diverse microbial consortium. This provides an environment conducive to the formation of a wide array of flavor compounds and nutrients in the vinegar. A variety of cereals serve as ingredients, including buckwheat, oats, sorghum, corn, wheat, barley, and rice [15]. Among them, Zhenjiang aromatic vinegar (ZAV) [17], Shanxi aged vinegar (SAV) [18], Sichuan Baoning vinegar (SBV) [19], and Yongchun aged vinegar (YCAV) [20] are representative traditional Chinese cereal vinegars. Different cereal vinegars have different raw materials and flavors. For example, Zhenjiang aromatic vinegar and Yongchun aged vinegar are based on glutinous rice, Shanxi aged vinegar is based on sorghum, and Sichuan Baoning vinegar is based on wheat. Figure 1 takes cereal as the entry point and summarizes the common varieties and raw materials of Chinese cereal vinegar.

### 2.1. Sorghum Vinegar

Sorghum is a principal raw material in the traditional brewing industry and is valued for its rich composition of starch, protein, fat, and tannins [21,22]. SAV is made of sorghum by starch saccharification, alcohol fermentation, acetic acid fermentation, fumigating, and aging. It is characterized by its distinct black color, thick texture, mellow and rich, and long sour taste. It gives the old vinegar its unique flavor and color of sour, fragrant, sweet, and fresh [9]. Furthermore, SAV exhibits significant antioxidant, antibacterial, and anti-inflammatory properties [23].

### 2.2. Rice Vinegar

The varieties of rice vinegar are abundant [24,25]. Among them, glutinous rice vinegar is made of high-quality glutinous rice through different fermentation methods. Studies indicate that fermented glutinous rice develops enhanced flavor complexity compared to traditional fermented rice [26]. The representative glutinous rice vinegars are ZAV and YCAV.

ZAV is produced from glutinous rice and wheat bran through a distinctive solid-state fermentation process. It is renowned organoleptically for being sour yet non-acerbic, fragrant with subtle sweetness, possessing a rich body and umami character, and exhibiting enhanced aromatic complexity with extended aging [27]. YCAV is made of glutinous rice, yeast, sesame, and other main raw materials by a unique liquid fermentation process. This unique production method imparts characteristic properties: brown and black color, a harmonious sweet-acid balance, mellow and refreshing, exceptional storage stability, and resistance to spoilage [28,29]. Zhejiang rosy vinegar (ZRV) is made of high-quality indica rice as raw material and fermented by natural mixed strains. The product has the characteristics of a delicate rose color, a soft and refreshing sour taste, and slightly fresh and sweet [30].

### 2.3. Wheat Vinegar

Wheat, a staple cereal crop providing approximately 18% of global daily dietary calories, is rich in protein, resistant starch, dietary fiber, and carotenoids and other nutritional values [31]. SBV is made of bran and wheat as raw materials, and the starter material is made of traditional Chinese medicine such as sand kernel, malt, hawthorn, cinnamon, and so on [32]. It is unique due to the addition of Chinese herbs and is considered to be the only medicinal Chinese vinegar. These herbs promote fungal proliferation in daqu during fermentation and contribute to the formation of unique aroma characteristics [33].

### 2.4. Buckwheat Vinegar

Buckwheat, a cereal plant belonging to the Polygonum family, possesses documented anti-diabetic, anti-inflammatory, and anti-cancer properties [34]. Flavonoids, phenolic acids, triterpenoids, and resistant starch in buckwheat are considered to be the main components [35,36]. SAV has developed a new product of buckwheat vinegar, which is fermented from buckwheat combined with traditional vinegar fermentation technology. This innovation retains the characteristic organoleptic qualities of traditional SAV while incorporating the distinctive nutritional benefits associated with buckwheat. Especially the effect of lowering blood lipid, lowering blood glucose, antioxidation, and softening blood [37].

### 2.5. Oat Vinegar

Oats contain high levels of protein (12.4–24.5%), dietary fiber, unsaturated fatty acids and phenolic compounds, *β*-glucan, and cellulose [38]. It has cholesterol-lowering and anti-diabetic effects [39,40]. Solid-state fermentation effectively preserves the comprehensive nutritional value inherent to oats [41]. Studies indicate that oat vinegar exhibits potent free radical scavenging capacity and inhibits lipid peroxidation [42]. Furthermore, oat vinegar demonstrates significantly stronger antioxidant activity compared to buckwheat vinegar [43].

### 2.6. Barley Vinegar

Highland barley, a low-utilization barley variety, contains nearly 80% carbohydrates, 11.5–14.2% protein, 3.7–7.7% *β*-glucan, 1.8–2.4% ash, and 4.7–6.8% lipids [44,45]. Zhang et al. [46] fermented sorghum with highland barley instead of SAV, they observed higher concentrations of volatile acids and esters in the resulting highland barley vinegar compared to SAV.

As one of the oldest and most widely used cereals in the world, barley is the fourth largest cereal after wheat, rice, and maize [47,48]. Metabolites such as organic acids and enzymes produced by microbial fermentation processes contribute to the higher nutritional value of barley. Fermented barley has the effects of lowering lipids [49,50,51], improving blood lipids [52], and glucose tolerance levels [53,54,55]. Researchers conducted acetic acid fermentation on lactic acid bacteria and barley fermented lactic acid bacteria barley wine (LBW) to obtain lactic acid bacteria-malt vinegar (LAB-MV). The characteristics of Lactic acid bacteria-malted vinegar (LAB-MV) during the fermentation process and its anti-hyperlipidemia effect were studied. It was found that the contents of organic acids, free sugars, ethanol and free amino acids in LAB-MV changed significantly. It significantly reduced lipid accumulation and total cholesterol levels in HepG2 cells. LAB-MV has significant properties of anti-hyperlipidemia and inhibition of cholesterol production [56].

### 2.7. Other Cereal Vinegar

Beyond common cereal vinegars mentioned above, there are also cereal vinegars brewing using corn, rice buds in the germinating state or wheat germ as raw materials. They can play a full role in the grain’s nutritional effects and strengthen the cereal vinegar’s function [37,57]. For example, researchers applied traditional Shanxi vinegar-making techniques to bitter buckwheat flour, oat bran, and black bean skin, producing coarse cereal vinegar powder with 8.53 g/100 g total acidity. This innovation diversifies cereal vinegar product lines and advances functional vinegar development [58].

While cereal vinegars are valued for their nutritional profile and distinctive flavor, current research disproportionately focuses on common cereal and fruit vinegars. Development of non-traditional cereal vinegars remains limited. Future innovations in the types of cereal vinegar can be strengthened to develop more multi-functional cereal vinegars to give full play to the nutritional benefits of grains and to meet consumers’ demand for healthy food.

## 3. Differences in Key Flavor Compounds of Traditional Chinese Cereal Vinegar

Vinegar, an acidic condiment, imparts unique sour, umami, and aromatic notes to foods. The flavor constituents of traditional cereal vinegar are composed of nonvolatile flavor constituents (taste) and volatile flavor constituents (aroma) [29,59]. Among these, organic acids and amino acids constitute the principal nonvolatile components in cereal vinegar.

### 3.1. Differences in Organic Acid Composition in Cereal Vinegar

Cereal vinegar is known for its unique sour taste, which is mainly derived from its rich organic acids, which not only give cereal vinegar its unique flavor but also promote appetite.

Organic acids constitute the most abundant and significant flavor compounds in cereal vinegar [60], and the variety of organic acids is much larger than that of fruit vinegar [61]. Organic acids in cereal vinegar are classified as volatile (e.g., acetic, propionic, butyric) or non-volatile (e.g., lactic, malic, tartaric, citric) [62]. Acetic and lactic acids are the major organic acids in cereal vinegar.

Among them, Acetic acid has the highest content of all volatile organic acids in cereal vinegar and has strong irritation. It has the effects of anti-inflammation, anti-obesity, regulating blood lipids, preventing ulcerative colitis, and maintaining intestinal immune homeostasis [63]. Acetic acid is considered a key factor in improving glucose uptake and reducing chronic disease risk in the long term [64,65]. Lactic acid, the major non-volatile acid, collaborates with malic, citric, and succinic acids to mitigate acetic acid’s sharpness, imparting softness and mellowness critical to product quality [66]. In addition to the above-mentioned organic acids with a relatively high proportion, gluconic acid is an essential non-volatile organic acid. It is a natural ingredient of fruits, plants, wine and honey [67]. It has been proposed as a quality parameter for traditional balsamic vinegar because it contributes to the aromatic characteristics and viscosity of the vinegar [68]. Some researchers conducted a study on the gluconic acid in different rose vinegars in China and found that the content of gluconic acid accounted for 0.48%. It primarily originates from acetic acid bacteria and *Aspergillus niger*, and its most common synthesis method is the oxidative fermentation of glucose by glucose oxidase [67].

Tong et al. [59] analyzed the organic acid composition and content across 17 bran-based vinegars, revealing a total organic acid range of 4100–7500 mg/100 mL. Overall, acetic and lactic acids accounted for more than 80% of the total organic acids in vinegar, making them the predominant organic acids with minimal variation between geographical regions. The types and concentrations of other organic acids varied greatly between vinegar products, and lactic acid and succinic acid were identified as characteristic organic acids in SBV. Within SAV, the content of most organic acids increased significantly during the acetic acid fermentation stage. Specifically, Quinic acid increased markedly from 0.67 g/L to 26.05 g/L, and citric acid content was significantly higher than that of other cereal vinegars [69]. These non-volatile organic acids neutralize the irritation of acetic acid and make SAV taste softer. YCAV was characterized by higher relative levels of acetic acid and fumaric acid [9]. Concentrations of other organic acids in YCAV were markedly lower than in the other three vinegars (SAV, SBV, ZAV), a characteristic attributed to its liquid-state fermentation process. The total amount of organic acids in SBV was the highest, which might be related to the fermentation process and environment. Furthermore, SBV displayed a distinct predominance of lactic acid over acetic acid, a feature significantly more pronounced than in other vinegars [70].

Analysis of organic acid profiles in SAV, ZAV, YCAV, and SBV revealed acetic and lactic acids as the main organic acids all four vinegars. The lactic acid content of solid-state fermented cereal vinegar was significantly higher than that of liquid-fermented cereal vinegar. Organic acid concentration directly correlated with total acidity, serving as a key indicator of product quality. Different fermentation materials and fermentation processes lead to great differences in the organic acids of cereal vinegar.

### 3.2. Differences in Amino Acid Composition in Cereal Vinegar

The content of free amino acids in cereal vinegar is rich. Due to the different raw materials and processes, the content and types of amino acids in cereal vinegar are also different. As essential flavor compounds, amino acids enhance palatability, impart taste complexity [71], and serve as primary sensory evaluation indicators [72]. Essential amino acids are indispensable nutrients for the human body and can only be acquired from food. Therefore, nutritional supplementation is also of great significance. Quantitative analyses reveal sweet and bitter amino acids predominate in most cereal vinegars, followed by umami-imparting varieties. Their collective interaction modulates and enhances overall flavor [73]. Cereal vinegars typically contain higher amino acid concentrations than fruit vinegars, with glutamate, alanine, valine, aspartate, lysine, and proline being most abundant. Some amino acids in cereal vinegar are derived from microbial metabolism, while the others come from the hydrolysis of proteins in the raw materials. Cereal vinegar can blend with the flavor of cereal vinegar and participate in the Maillard reaction to produce more flavor compounds [74].

Researchers studied the amino acids of SAV, ZAV, YCAV, and SBV. They discovered that the total amino acid content of SAV was higher, which was attributed to the higher bran protein content utilized in SAV [75]. Reduced bran usage in YCAV and ZAV corresponded with lower amino acid concentrations. While tryptophan was undetectable in all four vinegars and histidine absent in YCAV, other proteogenic amino acids were detected. For each vinegar, alanine, glutamic acid, phenylalanine, tyrosine, and valine were the most abundant amino acids [9].

### 3.3. Differences in Volatile Flavor Compounds in Cereal Vinegar

Volatile flavor compounds constitute the aromatic components of cereal vinegar, which is an important part of the flavor of cereal vinegar. These compounds impart distinctive aroma characteristics that directly influence consumers’ sensory perception and hedonic response [76]. As one of the important indicators of product quality and consumer acceptance, the aroma quality of cereal vinegar has received much attention [77,78].

Volatile flavor compounds have the characteristics of lower content and richer types. Volatile flavor compounds in cereal vinegar mainly include alcohols, esters, aldehydes, acids, ketones, etc. These compounds have floral, fruit, nut, and cream aromas [79,80]. Among the alcohols, ethanol is an important substrate for the aroma of cereal vinegar, and key alcohols include 2,3-butanediol, isoamyl alcohol, benzyl alcohol, and phenethyl alcohol [15]. However, excessive alcohol compromise sensory quality [81]. These alcohols originate from yeast-mediated sugar metabolism during alcoholic fermentation [82], subsequently undergoing esterification with acids in later stages [83]. Volatile acids represent a dominant aroma category in cereal vinegar, with acetic acid constituting the principal volatile component. Acetic acid, 3-methylbutyric acid, and caproic acid are the most abundant acidic compounds [15]. Esters constitute fundamental flavor constituents in cereal vinegar, characterized by fruity or floral aromas with pronounced volatility. They are the main components of the characteristic flavor of cereal vinegar, including ethyl ester, acetate and lactate ester [84]. Aldehydes are mainly produced by amino acid catabolism and microbial metabolism. Excessive aldehydes have a heavy, spicy taste, which harms cereal vinegar. Pyrazines contribute roasted-nutty aroma characteristics [85]. Furans usually have a caramel aroma, are mainly produced by the Maillard reaction, and their content tends to increase during the frying and aging of cereal vinegar [86].

Wang et al. [87] employed quantitative descriptive analysis (QDA) to characterize the sensory profile of ZAV, coupled with GC-IMS for volatile compound identification. Their analysis detected 28 volatile compounds across ZAV grades, categorized as: 6 aldehydes, 6 ketones, 7 alcohols, 3 esters, 3 pyrazines, and 3 miscellaneous compounds. Li et al. [88] used HS-SPME-GC/MS to analyze five samples of ZAV with different concentrations. They identified and quantified 40 volatile compounds, including acids, esters, alcohols, ketones, aldehydes, and other compounds. These compounds work together to create the unique aroma of ZAV. Zhou et al. [89] found that the formation of volatile acids in ZAV mainly occurred in the fermentation stage of acetic acid.

Some researchers studied the differences of volatile flavor compounds in SAV, ZAV, YCAV, and SBV. Fourteen esters, 7 pyrazines, 5 alcohols, 10 acids, 4 furans, 8 phenols, 10 aldehydes, 4 ketones, and 6 other volatile substances were identified. They identified 68 volatile compounds in total. Ethyl acetate, acetic acid, furfural, hexanoic acid, and benzaldehyde constituted common volatile compounds [9]. There are more varieties of phenols and ketones in SAV and ZAV [90], while the varieties of aldehydes in YCAV are significantly less. 2-methylpropyl acetate was exclusively detected in YCAV. Among all vinegars, esters and acids demonstrated the highest abundance. Furans were predominantly present in ZAV and SBV. Esters constituted the dominant volatile class (35.13–40.38%) after acids, substantiating their crucial contribution to aroma profiles [9]. Table 1 also summarizes the composition of key flavor substances and aromas of different cereal vinegars.

Cereal vinegars demonstrate substantial heterogeneity in aroma composition across different varieties. Moreover, even within the same vinegar type, volatile profiles exhibit significant divergence due to variations in fermentation process parameters and analytical methodologies employed during characterization.

## 4. Key Factors Affecting the Flavor of Cereal Vinegar

As a significant condiment, the flavor of cereal vinegar is affected by many factors. Including the environment, raw materials, microbial type (starter), and fermentation method [98]. Different conditions make the aroma composition of cereal vinegar in different regions of our country completely different. Table 2 summarizes the differences in raw materials, fermentation processes, and key microorganisms among different cereal vinegars.

### 4.1. Differences in Fermentation Materials of Different Cereal Vinegars

As the fundamental basis of cereal vinegar flavor formation, raw materials exert a decisive influence on the final product’s aroma and taste profile. Raw materials are the most important influencing factors. Different raw materials have different flavor compounds, and some flavor compounds will be retained in the cereal vinegar, which will affect the flavor of the vinegar. On the other hand, different raw materials contain different nutrients [104], and the difference in nutrients leads to the difference in the composition and concentration of the final metabolites, which in turn affects the flavor of cereal vinegar [10].

Zhenjiang aromatic vinegar (ZAV) primarily utilizes glutinous rice as its main substrate, supplemented by wheat bran and rice husk [105]. The protein component of glutinous rice provides nutrients for yeast proliferation during vinegar brewing. At the same time, it is broken down into free amino acids, which are used as precursors for microbial metabolism to produce the aroma compounds of vinegar. Amino acids and aroma compounds constitute the unique taste and aroma of ZAV, suggesting that these substances have a significant effect on the flavor of ZAV [10]. Li Xin et al. [102] respectively used glutamic rice and japonica rice as raw materials for fermentation through the ZAV fermentation process. They discovered that there was no significant disparity in the basic physical and chemical indices and organic acid indices of vinegar. However, slight variations in amino acid composition and substantial disparities in ester content were observed, resulting in markedly divergent volatile compound in the final products. Notably, glutinous rice-derived vinegar exhibited greater diversity and concentration of volatile flavor compounds. The key procedure in vinegar production is the transformation of alcohol into acetic acid by acetic acid bacteria.

Sorghum, the main raw material of SAV, is rich in nutrients such as protein, carbohydrate, fat, vitamins, and minerals [106,107]. It can provide energy and nutrition for the growth and reproduction of acetic acid bacteria, thereby promoting their growth and metabolism in the vinegar brewing process, thus producing unique flavor [99].

Highland barley contains various nutrients such as biologically active carbohydrates, polyphenols, minerals, vitamins, phenols, flavonoids, dietary fiber (8.16–21.46%) and *β*-glucans [108,109]. Highland barley vinegar is a type of solid-state fermented vinegar that has been extensively utilized in the Qinghai-Tibet Plateau of China. These nutrient matrices yield characteristic aroma compounds, including isoamyl acetate, ethyl octanoate, 1,2-propanediol, 3-methyl-1-butanol, and ethyl isovalerate, which serve as key flavor biomarkers [61].

### 4.2. Differences in the Fermentation Process of Different Cereal Vinegars

Due to the differences in geographical factors, although they are all Chinese cereal vinegars, cereal vinegars from different regions have their unique technological characteristics. Fermentation methodologies bifurcate into solid-state and liquid-state processes [106]. Among them, solid-state fermentation involves sequential saccharification, alcoholic fermentation, acetic acid fermentation, and aging—exemplified by Shanxi Aged Vinegar (SAV), Zhenjiang aromatic vinegar (ZAV), and Sichuan bran vinegar. Although they are all solid-state fermentation, they still have their unique techniques. SAV adopts a special fumigation process, half of the cupei (the fermented vinegar mash, termed cupei in Chinese) of acetic acid fermentation is fumigated over low heat, and the fumigation aroma is a typical flavor of SAV. The newly brewed vinegar is aged through sun-drying and winter ice scoting, which makes the vinegar flavor more intense [110]. The acetic acid fermentation of ZAV adopts the “solid-state layered fermentation” process. In an open environment, acetic acid bacteria are completely exposed to the air for acetic acid fermentation [111]. Sichuan bran bran vinegar is naturally inoculated. Saccharification fermentation, alcohol fermentation and acetic acid fermentation are carried out in the same pool [101]. YCAV and ZRV are prepared through liquid fermentation. Among them, YCAV is made through liquid deep fermentation and the raw materials are added in several batches. ZRV relies on natural strains in the environment for “flower formation” saccharification and is completed through a static surface fermentation method. And a unique three-side fermentation process is adopted, that is, saccharification fermentation, alcohol fermentation and acetic acid fermentation are carried out simultaneously [28,97].

Distinct processing methodologies generate differentiated flavor profiles in cereal vinegars. The flavor of cereal vinegar produced by traditional manual and modern technology is also quite different. Traditional solid-state brewing and closed solid-state brewing systems have large differences in physicochemical indexes and substance composition due to process differences [112], resulting in distinctive flavor qualities of the final product. The traditional solid cereal vinegar brewing system relies on a combination of natural microbial communities and environmental conditions to form a complex and unique flavor [113]. However, temperature and humidity fluctuations within grain substrates during summer fermentation may trigger heterogeneous microbial proliferation. This metabolic variability generates inconsistent flavor compound profiles, causing sensory instability in the final vinegar. These microorganisms may have produced different flavor compounds during metabolism, which caused instability of the flavor compounds and fluctuations in the flavor quality of the cereal vinegar [114]. Wang et al. [65] investigated the changing dynamics in the major quality indicators of vinegar during solid-state vinegar fermentation using a rotary drum bioreactor (RDB) and traditional fermentation (TF). The total acid content of raw vinegar produced in the RDB was 5.1% higher than that produced by TF, and the fermentation cycle was 6 days shorter. The main organic acids detected were acetic and lactic acid, and the total phenolic, flavonoid, ligustrazine, acetoin, and diacetyl levels tended to increase in the RDB. These results indicate that the RDB showed better fermentation performance than that of TF. Thereby demonstrating considerable potential for producing high-quality vinegar in industrial applications. Zhao et al. [115] studied and analyzed the chemical composition and functional characterization of traditional Zhenjiang aromatic vinegars (TZAV) and industrial Zhenjiang aromatic vinegars (IZAV) during aging. TZAV and IZAV samples had similar approximate compositions and organic acid content. However, TZAV samples represented more abundant phenolic compounds and stronger antioxidant capacity than IZAV samples.

### 4.3. Differences in Fermentation Environments of Different Cereal Vinegars

Traditional cereal vinegar is made by natural fermentation, and the complex microbial metabolism contributes to the vinegar flavor quality [72]. Compared to single-strain liquid fermentation, open multi-strain fermentation yields broader flavor profiles, albeit with heightened susceptibility to environmental perturbations that cause significant quality variations [116,117]. The variation of environmental factors in different seasons is the core factor leading to the differences in microbial community composition and structure [118].

Researchers employed HS-SPME-GC-MS and HPLC to analyze seasonal compositional variation in ZAV. The results showed that acetic acid content was highest in spring. The total lactic acid content was highest in winter. Succinic acid and pyroglutamic acid were highest in autumn. The total content of citric acid, tartaric acid, and oxalic acid was highest in summer. Compared to other free amino acids, the content of alanine and arginine in cupei (the fermented vinegar mash, termed cupei in Chinese) was higher in all seasons, with the highest alanine content in autumn and the highest arginine content in winter. The quality of ZAV in summer is different from that in other seasons. The volatile compounds in the fermentation process of ZAV in different seasons were detected, and it was found that the types of volatile flavor compounds in autumn were more abundant than those in other seasons. By the end of fermentation, the total content of volatile alcohols was highest in autumn, volatile esters and acids in summer, volatile aldehydes in winter, and volatile ketones in autumn [116]. In addition, high temperature can increase the content of polyphenols in vinegar and improve the flavor of vinegar [118]. High temperatures cause protein denaturation and the production of more free amino acids in vinegar [10]. Temperature significantly affects the total amount of ketones (mainly 3-hydroxybutanone, 2,3-butanedione, etc.) and heterocyclic substances in vinegar. Heterocyclic substances in vinegar were mainly generated through the Maillard reaction. The total amount of ketones in vinegar decreased significantly with increasing temperature, while the total amount of heterocyclic substances increased significantly [119].

Oxygen has an important effect on the fermentation process of cereal vinegar. During alcoholic fermentation dominated by yeast, the oxygen-rich upper layer of cupei (the fermented vinegar mash, termed cupei in Chinese) facilitates alcohol oxidation to acids, while anaerobic conditions in the lower layer promote ethanol production. By exposing the bottom of cupei, cupei can be in full contact with oxygen, thereby accelerating the conversion of alcohols to acids, esters, and volatiles, enriching the flavor of vinegar [10]. Zhang et al. [120] further quantified this spatial heterogeneity, revealing significantly higher acetic acid and ethanol but lower lactic acid in upper cupei, contrasted by elevated lactic acid concentration in lower cupei.

### 4.4. Differences in the Starter of Different Cereal Vinegars

Traditional Chinese solid-state brewing cereal vinegar is produced through an open and complex multi-strain mixed fermentation mode. The starter is the key to cereal vinegar, and the starter employed in different cereal vinegars varies.

ZAV fermentation employs specialized starters, such as the jiuqu that initiates the saccharification stage and enters the alcoholic fermentation process. Jiuqu is mainly made of rice flour, supplemented by Chinese herbal medicines and seed yeast, providing Aspergillus and yeast for saccharification, fermentation and alcohol fermentation. Maiqu is used in the alcoholic fermentation stage, mainly consisting of bran or crushed wheat. Seed pei is used in the acetic acid fermentation stage. It is cupei after being turned layer by layer at seven day. Provide *L. acetotolerans* and *A. jinshanensis* microorganisms for acetic acid fermentation. These starters can quickly start the fermentation process, ensuring the stability of the fermentation process and the flavor of the product [11]. The researchers investigated the effect of starters on microbial communities at different fermentation stages during the fermentation of ZAV. Jiuqu contributed 15% of the bacterial community and 10% of the fungal community at the beginning of the saccharification fermentation stage. Both raw materials and seed pei provided various microorganisms for the acetic acid fermentation stage, while the common dominant bacteria mainly originated from seed pei. This result suggests that seed pei is a necessary source of microorganisms with a balanced microbiota to start the acetic acid fermentation phase [11]. Daqu is the starter of SAV, which is produced through the natural fermentation of barley and peas in an open environment. Wei et al. [121] studied the dynamic changes of microbial community and volatile compounds and their relationship during the production of SAV. *Lactobacillus* and *Rhizopus* dominated the mature Daqu, with *Lactobacillus*, *Enterobacter*, *Bacillus*, *Aspergillus*, and *Rhizopus* exhibiting significant positive correlations with lactic acid and acetic acid accumulation. SBV is unique due to the addition of Chinese herbs and is considered to be the only medicinal Chinese vinegar. These herbs promote the proliferation of fungi in the fermentation process and contribute to the development of a unique aroma profile [94].

In recent years, the artificial construction of simplified synthetic bacterial flora with distinct functions and stable performance for the innovation of traditional processes has drawn extensive attention [122]. As starter, synthetic consortia ensure reproducible flavor quality [123], with biofortification proven effective in modulating microbial ecosystems and enhancing flavor profiles of traditional fermented foods [124,125]. Huang et al. [123] selected four strains of *A. pasteurianus*, *L. acetotolerans*, *Ac. Jinshanensis* and *L. helveticus* from the starter of ZAV during acetic acid fermentation. These four representative strains were synthesised into a starter for acetic acid fermentation. Compared with local starter, using synthetic microbial communities as starter has competitive advantages. It can rapidly start the fermentation process, improve substrate utilization, reduce the influence of seasonal fluctuations, and maintain product quality. Li et al. [126] engineered synthetic communities based on core functional microorganisms identified during Sichuan bran vinegar fermentation, successfully replicating its signature flavor profile while enhancing amino acid biosynthesis. The synthetic starter could replace the traditional starter to initiate acetic acid fermentation, and the product quality was similar to that of the traditional brewing process.

## 5. Mechanism of Flavor Compounds Formation

**Figure 2:**
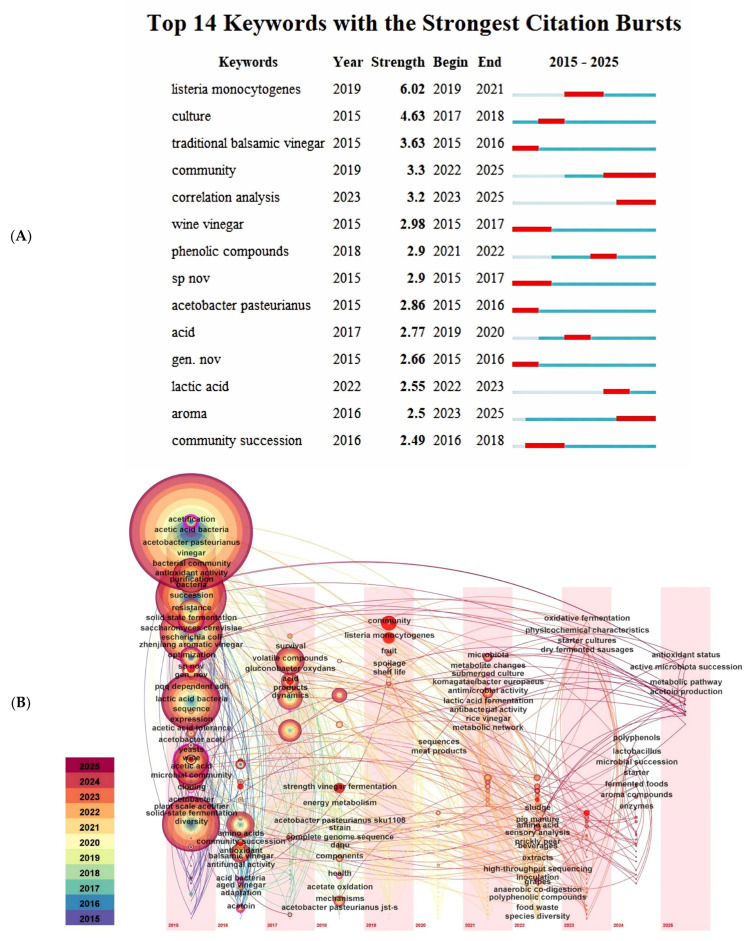
Time view of cited literature in research on microbial diversity and flavor matter changes in cereal vinegar. (**A**) Keyword emergent atlas. (**B**) Keywords time zone map.

Fermentation constitutes the core microbial bioconversion process in cereal vinegar production, encompassing three critical stages: starch saccharification, alcoholic fermentation, and acetic acid fermentation [127]. The diversity of microorganisms and their dynamic alterations have a significant impact on the quality and characteristics of products during the fermentation process [128]. As shown in Figure 2A,B, through Citespace cited literature clustering and comprehensive analysis of cited literature, In SCI database TS = ((vinegar) AND (bacteria) AND (Organic acid) OR (vinegar) AND (fungus) AND (Amino) acids) OR (vinegar) AND (microbial) AND (flavor)). It can be seen from Figure 2A that 14 key words were summarized during the period from 2015 to 2025. In the recent five years high frequency keywords for *listeria monocytogenes*, community, and the correlation analysis, phenolic come, acid, lactic acid and aroma. Figure 2B shows the distribution of each keyword in different time regions. In 2025, the research will mainly focus on antioxidant status, active microbiota success, etc. The key words such as acetification and vinegar have all been studied in the past decade.

### 5.1. Dominant Microorganisms in the Fermentation Process

The dominant microorganisms in different fermentation stages of different cereal vinegars are quite different. ZAV production demonstrates distinct bacterial and fungal community structures between fermentation phases. The dominant bacterial genera were *Acetilactobacillus*, *Acetobacter*, *Lactobacillus*, and *Pseudomonas* [111]. The relative abundance of two genera, *Acetobacter* and *Lactobacillus*, accounted for 85% of the total, while fungi included *Alternaria*, *Aspergillus*, *Rhizopus*, and *Saccharomyces* [11]. These microorganisms critically drive flavor compound biosynthesis (esters, ketones, alcohols) in ZAV. Nie et al. [99] studied the dynamic changes and diversity of microbial community succession during the whole fermentation process of SAV. Similarly, SAV exhibits dynamic microbial succession, where bacterial diversity surpasses fungal diversity. In addition, *Saccharomycopsis* and *Saccharomyces* exclusively dominate the alcoholic fermentation stage, whereas acetic acid fermentation initiates with *Lactobacillus predominance* (>50% abundance), followed by *Acetobacter* proliferation increasing from 17.79% to 29.77% during the middle and late stages of fermentation. These results indicate that *Lactobacillus* and *Acetobacter* as functional dominants, with *Acetobacter* demonstrating superior competitive fitness in community succession.

Analysis of the microbial composition of various cereal vinegars reveals that their production involves not only common microorganisms like acetic acid bacteria, lactic acid bacteria, and yeast, but also bacteria such as *Bacillus* and molds [72,126,129,130]. This diverse microbial community facilitates the synthesis of functional compounds like acetoin and tetramethylpyrazine in cereal vinegar, thereby conferring high nutritional value and health benefits to traditional Chinese cereal vinegars [131,132]. Acetic acid fermentation is the main stage for the formation and accumulation of flavor substances. Li et al. [102] studied the changes in microorganisms before and after the acetic acid fermentation stage of ZAV. They identified five key functional species, including *Komagataeibacter* sp., *Limosilactobacillus panis*, *Lactobacillus acetoolerans*. *Limosilactobacillus pontis* and *Acetilactobacillus jinshanensis* were identified as key functional microorganisms involved in the production of these unique metabolites by metatranscriptomics. Some researchers have conducted a combined analysis of the key flavor substances and microbial flora in Sichuan bran vinegar. The key microorganisms in the fermentation process were identified as *Brettanomyces bruxellensis* TYJ84j, *Pichia Kudriavzevii* TYJ7j, and *Lactobacillus plantarum* TYJ24. *Lactobacillus amylolyticus* TYJ23B, *Lactobacillus fermentum* TYJ23A, *Lactobacillus acetotolerans* TYJN10, *Acetobacter pasteurianus* TYJS4, *Lactobacillus amylovorus* TYJN8, *Acetobacter pomorums* TYJS6, *Clostridium beijerinckii* TYJD2, and *Lichtheimia ramose* TYJ1. Based on these strains, a synthetic starter culture was developed [126]. Zhang et al. [124] focused on the two dominant bacteria, *Lactobacillus helveticus* and *Acetobacter pasteurianus*. Using two native strains as raw materials, the fermentation process and flavor of cereal vinegar were targeted and regulated through different biological fortification strategies. Notably, sequential bioaugmentation specifically enhanced the contents of acetoin and tetramethylpyrazine (TMP).

Different fermentation conditions can affect the structure and succession of microbial communities. Therefore, there are significant differences in the microbial composition of cereal vinegars, which lead to the distinct flavor characteristics of different cereal vinegars.

### 5.2. Sources of Key Flavor Compounds in Cereal Vinegar

The environment in which cereal vinegar is traditionally fermented is a complex ecosystem. The characteristic flavor develops through microbial metabolic activities that utilize the fermentation matrix, decompose macromolecules, and release metabolites. After analyzing both microbial communities and flavor compounds in fermented foods enables the study of their correlations via statistical methods, elucidating microbial metabolic functions and flavor compound origins [133]. However, accurately distinguishing the sources of flavor compounds in cereal vinegar remains challenging due to their dynamic changes throughout the fermentation process.

*Acetobacter* and *Lactobacillus* are the main genera of organic acid metabolism in cereal vinegar, and some are directly derived from raw materials [134,135]. Wu et al. [136] constructed the flavor metabolic network of ZAV, revealing that amino acids in cupei derive either from raw material proteins/peptides or microbial synthesis. Notably, glutamate is synthesized by diverse vinegar microbiota (including *Actinobacteria* and *Lactobacillus*), while proline and arginine are predominantly produced by *Actinobacteria*, *Bacteroidetes*, *Bacillus*, and *Lactobacillus*. Alanine mainly arises from substrate protein/peptide degradation. Wang et al. [92] identified the aromatic active compounds in SAV and traced their origin throughout the production process. The main raw materials for SAV are lactone and aldehyde, whereas alcohols, esters, and phenolic compounds form through microbial activity during alcoholic fermentation. In addition, acids, alcohols, ketones, and esters are generated microbially during acetic acid fermentation, with most SAV flavor compounds originating from raw material degradation, amino acid/fatty acid metabolism, and Maillard reactions. Furthermore, huang et al. [11] studied the microbial community structure of the starter in each fermentation stage of ZAV, and constructed the substrate decomposition and flavor synthesis pathways in the fermentation process of seed pei. *Lactobacillus* and *Acetobacter* were the main metabolically active microorganisms in fermented grains. Carbohydrate metabolism and amino acid metabolism were the main metabolic pathways in seed pei.

### 5.3. Formation Pathways of Key Flavor Compounds of Cereal Vinegar

Advancements in sequencing technology have enabled extensive application of omics approaches based on microbial genetic information in cereal vinegar research. Recognition is growing regarding the importance of integrated methodologies, where multi-omics techniques combined with bioinformatics analysis provide a robust foundation for exploring fermentation processes. This integration facilitates systematic investigation of microbial community-flavor relationships and their formation mechanisms during fermentation [137,138,139].

**Figure 3:**
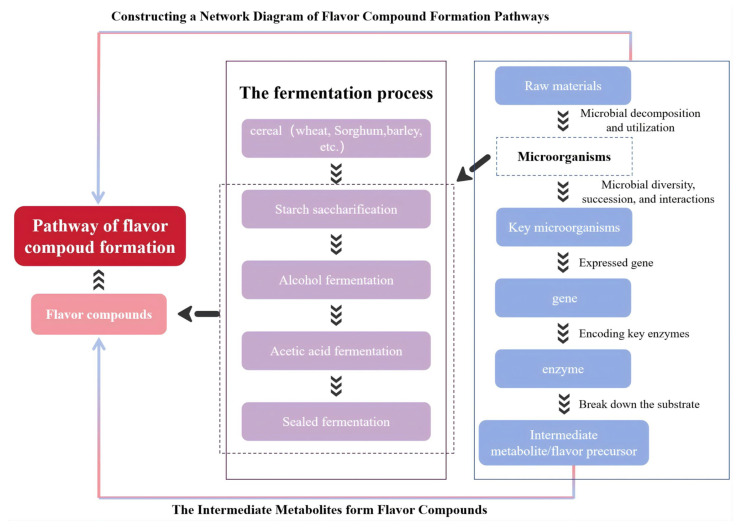
Research methods for the formation of flavor compounds in natural fermented vinegar.

Figure 3 summarizes current methodologies for elucidating flavor compound formation pathways. Fermentation is the process in which microorganisms decompose the matrix in raw materials into various flavor substances. The microbial community of fermentation has a significant impact on the synthesis of flavor substances. The metabolic pathways of different microbiota, microbiota diversity and microbiota interactions will ultimately affect the flavor characteristics. Analysis of microbial community dynamics during fermentation reveals stage-specific microbial succession and its correlation with flavor formation [140]. Microorganisms regulate enzymes through genes to form flavor substances. The functional genes of microorganisms were annotated, the enzymes related to flavor formation were screened, and the functional microorganisms related to flavor formation and substrate degradation in the fermentation process were identified. The pathways of flavor formation were further analyzed, and the metabolic network of flavor compounds was constructed [72].

Flavor compounds primarily originate from metabolic pathways including the TCA cycle, amino acid metabolism, fatty acid metabolism, carbohydrate metabolism, and nucleotide metabolism [141]. Organic acid biosynthesis—mediated predominantly by acetic acid bacteria, lactic acid bacteria, and yeast—progresses through the tricarboxylic acid cycle, citrate pyruvate cycle, and lipid oxidation [142]. Liu et al. [95] reconstructed lactic and acetic acid metabolic networks in SBV using metagenomics. The major biosynthetic pathway of lactate involves the conversion of pyruvate to D-lactate and L-lactate by D-lactate dehydrogenase and L-lactate dehydrogenase, respectively. The main microorganisms included *Acetilactobacillus jinshanensis*, *Limosilactobacillus*, and *Limosilactobacillus pontis*. Acetic acid synthesis primarily involves the conversion of pyruvate to acetyl-P by pyruvate oxidase and then to acetic acid by acetate kinase. *Lactobacillus amylovorus*, *Limosilactobacillus* sp., *Lactobacillus acetotolerans*, *Limosilactobacillus pontis*, and *Acetobacter pasteurianus* are the main producers. It has been shown that pyruvate is a core compound in the organic acid metabolic network. The metabolic pathway of acetate, which has a regulatory function on the metabolism of other organic acids, plays a key role in this network [8]. Liu et al. [143] studied the phenolic substances of SAV and found that phenolic acid was the key phenolic substance in the brewing of SAV. Phenol formation involves biochemical reactions (degradation, oxidation, decarboxylation, hydroxylation) primarily mediated by *Lactobacillus* and *Acetobacter*—dominant genera in both physicochemical parameter shifts and phenolic transformation. In fermented foods, Microorganisms can use these primary metabolites to generate secondary metabolites, which can be further broken down to produce volatile compounds [141]. Volatile flavor compounds are mainly produced during fermentation through different reactions, including caramelization, Maillard reaction, thermal degradation, lipid oxidation, and degradation of major and minor components (proteins, vitamins, pigments, sugars, ribonucleotides, etc.) [144].

The variations in the environment, raw materials, starter, and fermentation processes in different regions will result in certain disparities in the structure and succession of microbial communities. These are the key factors contributing to the differences in the flavor and quality of various cereal vinegars. Therefore, elucidating the structure and succession of complex microbial communities during cereal vinegar fermentation is the key to understanding the flavor formation mechanism of cereal vinegar.

## 6. Conclusions and Prospects

Traditional Chinese cereal vinegar occupies an important position in the food industry because of its distinctive flavor and abundant nutritional components. The flavor of cereal vinegar is mainly composed of organic acids, amino acids, alcohols, esters, aldehydes, ketones, and other compounds. Flavor characteristics vary substantially across vinegar types due to differences in raw materials, fermentation processes, and microbial community structures. Flavor compound generation is primarily governed by microbial metabolism, whereby microorganisms convert carbohydrates, proteins, and other substrates into diverse flavors and nutrients through complex metabolic pathways. We can utilize multi-omics techniques to gain a deeper understanding of the interaction between microbial communities and flavors. And reveal the metabolic pathways and regulatory mechanisms of key flavor substances. Exploration of alternative grains (barley, oat, buckwheat) or functional ingredient incorporation may facilitate novel vinegar development.

At present, although significant progress has been made in the research on the flavor of cereal vinegar. Current research faces limitations including restricted vinegar varieties and incomplete understanding of flavor compound differentiation and formation mechanisms. With the continuous increase in people’s attention to food health, it is of great significance to explore the health benefits of cereal vinegar and develop functional cereal vinegar. In conclusion, the research into traditional Chinese cereal vinegar has a broad scope for further development in terms of flavor, nutrition, and health functions.

## Figures and Tables

**Table 1:** Composition and aroma of key flavor compounds of different cereal vinegars.

Varieties	Key Flavor Compounds	Aroma	References
Shanxi Aged Vinegar (SAV)	acetic acid, furfuryl alcohol, 2,3-dimethylpyrazine, lactic acid, phenol, propyl acetate, ethyl propionate, and 2(5H)-furanone	Acidity, alcohol, orange peel, and yeast aromas	[91,92,93]
Sichuan Baoning Vinegar (SBV)	Lactic acid, 2-hydroxy-3-butanone acetate, butyrolactone, furan-2-aminoformaldehyde, acetic acid and 3-oxobutane-2-yl acetate	Fruity, sweet, baking, spicy, and woody	[94,95]
Zhenjiang Aromatic Vinegar (ZAV)	Acetic acid, furfural, aldehyde ketone, ethyl acetate, 3-methylbutyric acid, tetramethylpyrazine, isobutyric acid, ethyl phenylacetate, 3-hydroxy-2-butanone, 2, 3-butanedione, 2-acetofurfural, tetramethylpyrazine, phenyl ethyl ester, and phenylethanol	Caramel and buttery fruity, smoky, bran, and almond flavors	[89,96]
Yongchun Aged Vinegar (YCAV)	Isobutyl acetate, 2-acetoxy-3-butanone, and heptanoic acid, whereas octanoic acid, 2-dodecanol, butyric acid, styrene, ethyl caproate, and 1-nonanol	Aromas of fruit, ester, jackfruit, apple, banana, and keto	[29,84]
Zhejiang Rosy Vinegar (ZRV)	Acetic acid, lactic acid, ethyl acetate, and acetoin	Fruit, cream, butter, and caramel	[97]

**Table 2:** Differences in raw materials, fermentation process and key microorganisms of different cereal vinegar.

Varieties	Raw Materials	Fermentation Process	Key Microorganisms	References
Shanxi Aged Vinegar (SAV)	Sorghum, bran	Solid state fermentation (Use daqu instead of grain; Special fumigation technique; sun-drying and winter ice scoting)	*Acetobacter* (50.9%), *Lactobacillus* (47.9%), *Saccharomyces*	[99]
Sichuan Baoning Vinegar (SBV)	Bran, wheat, glutinous rice	Solid state fermentation (Fermentation in the same pool)	*Acetilactobacillus*, *Lactobacillus*, *Limosilactobacillus*, *Acetobacter*, *Weizmannia*, and *Lactiplantibacillus*	[19,100,101]
Zhenjiang Aromatic Vinegar (ZAV)	Rice, rice husk, bran	Solid state fermentation (solid-state layered fermentation)	Bacteria: *Lactobacillus*, *Acetobacter* Fungi: Moulds and yeasts	[11,102]
Yongchun Aged Vinegar (YCAV)	Glutinous rice and sesame seeds	Liquid fermentation (Liquid deep fermentation)	*Bacillus*, *Rhodococcus*, *Saccharomycelales* and *Pichia membranifaciens*	[28,84]
Zhejiang Rosy Vinegar (ZRV)	Indica rice	Liquid fermentation (Three-sided fermentation)	*Acetobacter*, *Lactobacillus*	[97,103]
